# Idiopathic hypereosinophilic syndrome associated with multivessel coronary artery ectasia: a case report

**DOI:** 10.11604/pamj.2025.51.103.47861

**Published:** 2025-08-21

**Authors:** Mehdi Ayoub Laaroussi, Nabil Laktib, Zouhair Lakhal, Najat Mouine, Aatif Benyass

**Affiliations:** 1 Department of Cardiology, Mohammed V Military Instruction Hospital, Mohamed V University, Rabat, Morocco

**Keywords:** Idiopathic hypereosinophilic syndrome, coronary artery ectasia, eosinophilia, case report

## Abstract

We report a rare case of idiopathic hypereosinophilic syndrome (iHES) associated with extensive multivessel coronary artery ectasia (CAE), a vascular complication seldom described in this condition. A 56-year-old man with known iHES presented with progressive exertional chest pain. Coronary angiography revealed diffuse ectasia of all major epicardial coronary arteries, with a critical stenosis in the mid-right coronary artery. Secondary causes of eosinophilia were excluded, and no clinical or serological evidence of vasculitis or connective tissue disease was found. The patient was treated with corticosteroids, immunosuppressants, and dual antiplatelet therapy, with a favorable outcome. This case illustrates the potential for eosinophil-mediated vascular injury in iHES and emphasizes the need for comprehensive cardiovascular assessment in patients with persistent hypereosinophilia, even in the absence of systemic symptoms. Increased awareness of this underrecognized manifestation may help guide timely diagnosis and management.

## Introduction

Idiopathic hypereosinophilic syndrome (iHES) is a rare multisystemic disorder defined by persistent, unexplained peripheral eosinophilia and organ damage in the absence of secondary causes [1]. Cardiac involvement is one of the most frequent and severe complications, typically affecting the endocardium and myocardium [2]. In contrast, coronary artery abnormalities, such as ectasia or aneurysms, remain exceedingly rare in this setting [1]. The underlying mechanisms behind coronary vasculopathy in iHES remain incompletely understood, but may involve direct endothelial toxicity from eosinophil granule proteins, immune-mediated vasculitis, and chronic inflammatory remodeling [2]. Only a few cases have described diffuse or focal coronary dilation in patients with iHES, usually in the absence of conventional cardiovascular risk factors or significant atherosclerosis [3]. We report a rare case of multivessel coronary artery ectasia in a patient with long-standing iHES, in whom extensive diagnostic workup excluded other causes of vascular abnormalities.

## Patient and observation

**Patient information:** a 56-year-old North African male, with a four-year history of idiopathic hypereosinophilic syndrome (iHES), presented for evaluation of progressively worsening exertional dyspnea and intermittent chest discomfort over the past six months. He had been diagnosed with iHES in 2020 following persistent eosinophilia >1500/µL and systemic symptoms including arthralgia and fatigue. At diagnosis, extensive workup excluded secondary causes such as parasitic infection, autoimmune disease, and hematologic malignancy. He had no significant family history of cardiovascular or autoimmune disease. Past treatment included corticosteroids and hydroxyurea, with partial hematologic response. The patient did not smoke, consumed alcohol occasionally, and had no relevant psychosocial or genetic factors.

**Timeline of current episode:** the patient was diagnosed with idiopathic hypereosinophilic syndrome (iHES) in 2020 following the discovery of persistent eosinophilia (>4000/µL) associated with systemic symptoms such as arthralgia and fatigue. Despite treatment with corticosteroids and hydroxyurea, his eosinophil counts remained chronically elevated over the following years. In late 2023, he developed progressive exertional dyspnea and chest discomfort, which led to his referral to the cardiology department in early 2024 for further evaluation. A chronological summary of the patient's clinical course is presented in Table 1.

**Table 1:** timeline of clinical events in the present case of idiopathic hypereosinophilic syndrome with coronary artery ectasia

Timepoint	Clinical event
2020	Initial diagnosis of iHES with eosinophil count >4000/µL
2020-2023	Treatment with corticosteroids and hydroxyurea; partial control of eosinophilia
Late 2023	Onset of exertional dyspnea and chest discomfort
Early 2024	Presentation to cardiology service and diagnostic evaluation

**Clinical findings:** on presentation, the patient was hemodynamically stable. Cardiovascular examination revealed a regular heart rhythm and a soft systolic murmur over the aortic area. There were no signs of heart failure or vasculitis (e.g., no purpura, Raynaud phenomenon, or neuropathy). The remainder of the physical examination was unremarkable.

**Figure 1:**
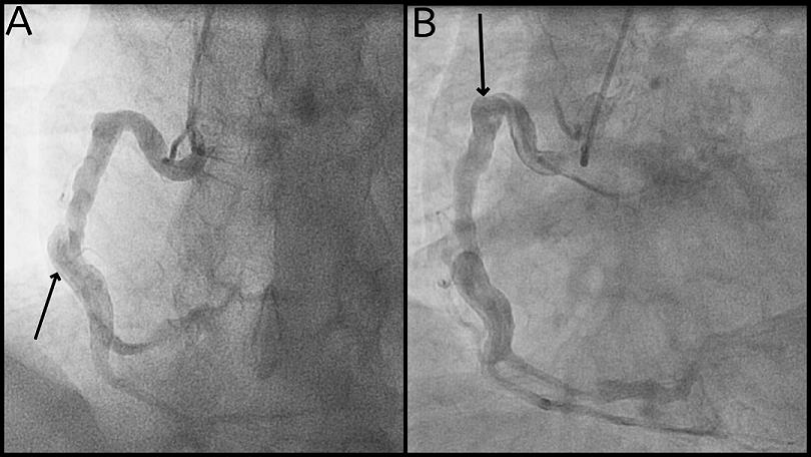
selective coronary angiography showing diffuse ectasia of the right coronary artery

**Diagnostic assessment:** laboratory tests confirmed persistent hypereosinophilia (3.5 × 10^9^/L). Inflammatory markers (CRP, ESR), renal function, and cardiac enzymes were normal. Autoimmune testing including ANA, ANCA, and complement levels was negative. Serum tryptase and FIP1L1-PDGFRA testing were unremarkable. Chest X-ray showed a prominent aortic contour without cardiomegaly. ECG revealed sinus rhythm with left anterior hemiblock and T wave inversions in inferior leads. Transthoracic echocardiography showed preserved left ventricular function without valvular abnormalities or segmental wall motion anomalies. Coronary angiography revealed diffuse coronary artery ectasia involving the left anterior descending, circumflex, and right coronary arteries, with a critical stenosis in the mid-right coronary artery (Figure 1). No atherosclerotic calcifications were noted. Imaging ruled out connective tissue disorders or inflammatory aortitis. No access or technical limitations were encountered during diagnostics.

**Diagnosis:** the final diagnosis was multivessel coronary artery ectasia in the context of idiopathic hypereosinophilic syndrome. Other potential causes such as atherosclerosis, vasculitis, and genetic syndromes were excluded based on clinical and laboratory workups. The condition was considered a vascular complication of chronic eosinophilic inflammation. Prognosis was considered stable under appropriate medical management, although with potential risk of thrombotic complications.

**Therapeutic interventions:** the patient was initiated on dual antiplatelet therapy (aspirin 100 mg/day and clopidogrel 75 mg/day). His immunosuppressive regimen for iHES (prednisone 20 mg/day and hydroxyurea 500 mg/day) was continued without modification. Statins and beta-blockers were not prescribed due to the absence of conventional cardiovascular risk factors. No surgical or interventional procedure was indicated at this stage.

**Follow-up and outcome of interventions:** at 3-month follow-up, the patient reported significant improvement in exertional symptoms and no recurrent chest pain. Repeat eosinophil counts remained elevated but stable under medical therapy. He showed good adherence to prescribed medications with no reported side effects. Follow-up imaging is planned to monitor the progression of ectasia.

**Patient perspective:** the patient expressed gratitude that his cardiac symptoms were taken seriously and attributed to his chronic condition. He stated that he was unaware that eosinophilia could affect the heart and was relieved to avoid invasive interventions. He is motivated to continue treatment and attend regular follow-up visits.

**Informed consent:** written informed consent was obtained from the patient for publication of this case and associated clinical data.

## Discussion

Idiopathic hypereosinophilic syndrome (iHES) is a rare hematologic disorder characterized by persistent eosinophilia exceeding 1,500/μL for more than six months and associated organ damage in the absence of identifiable secondary causes [3]. Cardiac involvement is among the most frequent and severe manifestations, typically presenting as Löffler endomyocarditis, valvular fibrosis, or intracardiac thrombi. In contrast, coronary artery involvement—particularly coronary artery ectasia (CAE) or aneurysm-is exceedingly rare and poorly characterized [1]. CAE is observed in approximately 1-5% of coronary angiographies, usually in the context of atherosclerosis [4]. In iHES, however, coronary vasculopathy has been described only in isolated case reports. Notably, Kang *et al*. (2023) reported a giant right coronary artery aneurysm with thrombosis in an iHES patient despite well-controlled eosinophil counts [3]. Similar cases support the link between chronic eosinophilia and coronary aneurysm formation [5]. These rare reports suggest that eosinophil-mediated vascular injury, even in the absence of systemic vasculitis, may contribute to coronary wall remodeling. Coronary aneurysms in iHES may remain silent or present with acute coronary syndromes such as angina or myocardial infarction due to thrombotic occlusion [3-6]. Some patients are entirely asymptomatic, with aneurysms detected incidentally on imaging. Regardless of presentation, the prognosis can be serious, with risks of thrombosis, rupture, or distal embolization. Given this potential, early and thorough cardiovascular imaging should be considered in patients with iHES, particularly when vascular symptoms or abnormalities are suspected [6].

Eosinophils contribute to vascular injury through the release of cytotoxic granule proteins such as major basic protein (MBP) and eosinophil cationic protein (ECP), which induce endothelial damage, activate platelets, and impair natural anticoagulant pathways [4]. These mediators promote a prothrombotic and proinflammatory vascular environment. Histopathological studies in hypereosinophilic syndrome (HES) have demonstrated eosinophilic infiltration and granule deposition within the vessel wall, often associated with necrosis and medial degeneration [7]. Such mechanisms not only underlie eosinophilic vasculitis but also predispose to vascular remodeling, dissection, and aneurysm formation [1]. In this context, coronary artery involvement likely represents a continuum of systemic eosinophil-driven vasculopathy, with eosinophil degranulation acting as a central pathophysiological driver.

Eosinophilic cardiac injury in hypereosinophilic syndrome (HES) may remain subclinical until advanced stages, which has led experts to recommend early and comprehensive cardiovascular imaging as soon as the diagnosis is established [1]. Cardiology guidelines particularly emphasize the utility of biomarkers and imaging during the asymptomatic phase. Bondue *et al*. (Heart 2022), for instance, advocate for the use of serum troponin and cardiac magnetic resonance imaging (CMR) in the early detection of silent myocardial involvement [8]. While transthoracic echocardiography often fails to detect early-stage disease, CMR can reveal myocardial edema and fibrosis prior to symptom onset. The authors specifically note that at this stage, related to subendocardial eosinophilic infiltrates, elevation of the biomarker and cardiac MRI are the best tools for diagnosis. As the disease progresses, echocardiography becomes more informative in identifying mural thrombi, valvular dysfunction, or endocardial fibrosis. In one study published in the Journal of Cardiovascular Magnetic Resonance, approximately 80% of HES patients referred for suspected cardiac involvement exhibited abnormal findings on CMR, including nonischemic late gadolinium enhancement, myocardial edema, or endomyocardial fibrosis, underscoring the value of early CMR screening [9]. Coronary angiography is not routinely performed but becomes indicated in the presence of ischemic symptoms or electrocardiographic changes. Most reported cases of coronary aneurysm in HES have been identified via angiography following clinical suspicion. Additional vascular imaging with chest CT or MR angiography may be warranted in patients with marked eosinophilia to screen for systemic aneurysms. Torcida *et al*. have proposed that such screening could be clinically valuable, even though no formal consensus exists [6]. They emphasize that medium- and large-vessel aneurysms, although uncommon, represent serious complications of HES, justifying individualized follow-up imaging strategies in selected cases.

Overall, there are no formal imaging guidelines specific to hypereosinophilic syndrome (HES), prompting clinicians to adopt a low threshold for cardiovascular investigation. Expert recommendations emphasize early cardiac evaluation in all patients with HES, including ECG, troponin levels, and echocardiography or cardiac magnetic resonance imaging (CMR), to detect endomyocardial involvement [6-8]. Additional vascular imaging should be considered when there is any suspicion of vasculitis or arterial aneurysm.

## Conclusion

Multivessel coronary artery ectasia occurring in the context of idiopathic hypereosinophilic syndrome is an exceptionally rare manifestation of eosinophil-driven cardiovascular disease. It illustrates the capacity of chronic eosinophilic inflammation to affect epicardial coronary arteries even in the absence of conventional cardiovascular risk factors. Diagnosis requires a high degree of clinical suspicion, particularly in patients presenting with unexplained chest symptoms and persistent hypereosinophilia. The key takeaway from this case is the importance of early cardiovascular imaging in patients with iHES, as timely detection of vascular involvement may alter management and reduce the risk of thrombotic complications.
